# Intraoperative airway management for patients with tracheal tumors: A case series of 37 patients

**DOI:** 10.1111/1759-7714.14181

**Published:** 2021-10-09

**Authors:** Rong Gao, Xiaolan Gu, Shuai Zhang, Shuliang Ma, Lin Xu, Ming Li, Lianbing Gu

**Affiliations:** ^1^ Department of Anesthesiology Nanjing Medical University Affiliated Cancer Hospital & Jiangsu Cancer Hospital & Jiangsu Institute of Cancer Research Nanjing China; ^2^ Department of Thoracic Surgery, Jiangsu Key Laboratory of Molecular and Translational Cancer Research Nanjing Medical University Affiliated Cancer Hospital & Jiangsu Cancer Hospital & Jiangsu Institute of Cancer Research Nanjing China

**Keywords:** airway management, anesthesia, tracheal resection and reconstruction, tracheal tumor

## Abstract

**Background:**

Tracheal tumors are rare. The aim of this case series was to investigate airway selection during radical surgery for patients with tracheal tumors.

**Methods:**

Here, we performed a retrospective case review of patients with tracheal tumors who underwent tracheal surgery in our center. A total of 37 cases, including 26 patients with primary tracheal tumors and 11 cases with advanced thyroid cancer, were enrolled into the study. Baseline characteristics and differential prognosis of included patients were estimated. We summarize the strategies for intraoperative airway selection and analyze the risk factors associated with delayed extubation.

**Results:**

There is a trend for primary tracheal tumors to appear toward the upper (9 of 26) and middle third (9 of 26) of the trachea, followed by the lower third airway (8 of 26). Advanced thyroid cancers occur most frequently in the upper trachea (7 of 11) and then the middle trachea (4 of 11). All primary and secondary patients underwent R0 resection. Minor histological subtypes were found to correlate with a poor prognosis. Extracorporeal support and tracheotomy intubation were applied in high‐risk cases, and a total of 32 patients achieved intrathoracic intubation during the surgical process. Intensive care unit (ICU) delay (>1 day) was observed among 25 patients, which were not enriched in cases who underwent cross‐field endotracheal intubation. Additionally, temporal suboptimal oxygenation (SpO2 < 95%) was an independent risk factor of ICU delay.

**Conclusions:**

Airway selection plays an important role in successful tracheal surgery, and an appropriate ventilation routine depends on the patient and a surgical process which is safe and effective.

## Graphical abstract

We reviewed 37 patients with tracheal tumors in our center and summarized the tumoral features, anesthetic considerations, and airway management during tracheal surgery. This study indicated a favorable curative effect of tracheal tumors is achievable and suggests the safety of personalized intraoperative intubation.

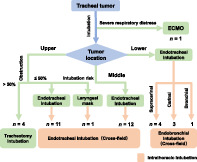

## INTRODUCTION

Malignant tracheal tumors are rare. Primary tracheal tumors have been found to harbor heterogeneous features on histopathology,^1^ and carcinoma originating in the thyroid and esophagus have been reported to contribute to secondary tracheal tumors with airway extrusion.^2^, ^3^ Severe tracheal obstruction has been observed among patients with tracheal tumors, which not only induce pulmonary symptoms but also lead to a difficult surgical procedure and airway management, especially for patients with lower tracheal tumors.^4^ At present, tracheal resection and reconstruction is a relatively safe but inherently complex treatment method,^5^ which is associated with high surgical success and postoperative complication rates at the same time.^6^ Tracheal resection and reconstruction is the preferred treatment for experienced centers, and our center is one of the earliest thoracic centers to perform tracheal surgery in China.^7^, ^8^ It is well known that the airway is shared by both surgeon and anesthesiologists during tracheal surgery, and therefore close communication is needed, in order to benefit the surgical procedure and improve ventilation safety.

Multidisciplinary strategies for intraoperative ventilation during tracheal resection and reconstruction are technical challenges for anesthesiologists. Few studies have previously focused on airway management of tracheal operation systematically. Krecmerova et al. and Schieren et al. reported the application of laryngeal mask airway in tracheal surgery.^9^, ^10^ A previous review of the literature reported all ventilation methods for tracheal surgery from 59 publications, most of which were case reports.^11^ In summary, diverse tumor locations and corresponding surgical strategies resulted in distinct intraoperative airway selections during tracheal resection and reconstruction,^1^ and therefore operation teams performed different strategies for airway management due to the lack of unified guidelines.

In this case series, we retrospectively summarized intraoperative ventilation strategies for patients who underwent tracheal surgery in our single center over the past 10 years. We integrated baseline characteristics and airway selections for patients with upper, middle, and lower tracheal tumors, respectively, and estimated the long‐term prognosis of different histological subtypes and further analyzed potential risk factors of delayed extubation.

## METHODS

This study has been registered with the institutional review board of Jiangsu Cancer Hospital. A retrospective case review was performed of all tracheal tumor cases who underwent tracheal surgery in the department of thoracic surgery from January 2010 to October 2020. Anesthetic data for general anesthesia, tracheal intubation, and mechanical ventilation were gathered from electronic medical records and the database of the department of anesthesiology. The publication of this single‐center case series was approved by the Ethics Committee of Jiangsu Cancer Hospital, and the written informed patient consent was waived.

### Preoperative evaluation

All patients were symptomatic, and cough, dyspnea, hemoptysis, and fever caused by tracheal obstruction were frequent manifestations among cases in this study. Cardiac and pulmonary function were evaluated preoperatively using two‐dimensional echocardiogram, electrocardiogram, and pulmonary function tests. Cardiac or pulmonary comorbidities should be painstakingly evaluated to assess any potential surgical risk. Preoperative evaluation also consisted of chest radiography and chest computed tomography, which were a theoretical basis for the following surgical strategy and airway management. To group those cases with tracheal tumors, the upper third of the trachea was defined as extrathoracic trachea according to a previous study,^12^ and the lower airway included the lower third trachea and bronchi.^13^, ^14^ Imaging examination of the brain and bone were also routinely performed to exclude potential distant metastasis. Bronchoscopy was always applied preoperatively to estimate the extent of tracheal resection, especially in patients with severe tracheal obstruction and carinal involvement.^15^ Baseline characteristics, including tumor location, level of tracheal obstruction, and brittle texture, were confirmed with bronchoscopy, and no therapeutic bronchoscopy was performed.

### Anesthetic considerations and airway management

Noninvasive monitoring and total intravenous anesthesia was applied in all cases. Penehyclidine, propofol, fentanyl, and midazolam were used for anesthetic induction, and rocuronium bromide was applied as a muscle relaxant. Ventilation with tracheal intubation was applied under the guidance of a fibrobronchoscope to avoid contact with the tracheal tumor. An anesthesia machine was used as a mechanical ventilator to maintain the blood oxygen saturation (SpO2) above 95%. Any temporal decline in blood oxygen saturation (SpO2 < 95%) was recorded when keeping oxygen saturation above 95% during the surgical process, as indicated in previous studies.^16^, ^17^

We performed diverse airway managements of ventilation and intubation, including tracheotomy intubation, laryngeal mask, and endotracheal intubation, on patients included in this study. After circumferential dissection, cross‐field ventilation of intrathoracic intubation was used to maintain the oxygen supply during the surgical procedure. For the case in which video‐assisted thoracic surgery (VATS) was performed, the fifth intercostal space was used for the transition of intubation. Venoarterial extracorporeal membrane oxygenation (VA‐ECMO) was performed on the case with severe tracheal obstruction. and anticoagulants are required to prevent circuit thrombosis.^18^ In brief', peripheral VA mode of femoral artery–vein catheterization was performed, and initial blood flow was maintained at arterial pressure of 65 mmHg; the ratio of air flow to blood flow was set at 1:1 with an oxygen concentration of 100%, which was then regulated according to arterial blood gas analysis. The median length of postoperative intensive care unit (ICU) stay was one day, and ICU stay was therefore defined (>1 day) as ICU delay.

### Surgical treatment

Thirty‐four patients underwent circumferential resection of the trachea and consequently end‐to‐end anastomosis for which four to seven tracheal rings were resected. Among these cases, the thyroid was removed with the upper trachea in patients with an advanced thyroid tumor invading the airway, and median sternotomy was performed in cases with middle or lower tracheal tumors. Tracheal reconstruction was performed on three cases with carinal involvement as previously described.^8^ In brief, the right or left fifth intercostal space was used to enter the chest and access the carina by means of posterolateral thoracotomy. Four incisions were made preoperatively in the case who underwent VATS resection, including one camera port, two operative ports, and one intubation port for intrathoracic intubation. After anastomosis, the saline solution was used to test the evidence of air leaks, and bronchoscopy was also conducted to check the integrity of the anastomotic stoma. Upon completion of the operation, an additional “guardian” stitch between the chin and chest was placed to avoid excessive tension for 2 weeks.^8^, ^16^ A total of six cases experienced postoperative complications, including anastomotic fistula (*n* = 3), anastomotic stenosis (*n* = 1), and vocal cord paralysis (*n* = 2).

### Statistical analysis

Statistical analyses was performed using R (v3.5.1). Baseline characteristics were indicated as number of categorical variables. Categorical variables were compared using the Pearson's chi‐square test or Fisher's exact test. Analysis of overall survival (OS) was performed using the Kaplan–Meier method, and the log‐rank test was used to compare survival curves. A permutation test procedure for inference in logistic regression was performed using R package glmperm.^19^ All *p*‐values were two sided with a significance level of 0.05. Other figures were generated using the R package ggplot2 and RColorBrewer.

## RESULTS

### Characteristics and prognosis of patients with tracheal tumor

**FIGURE 1:**
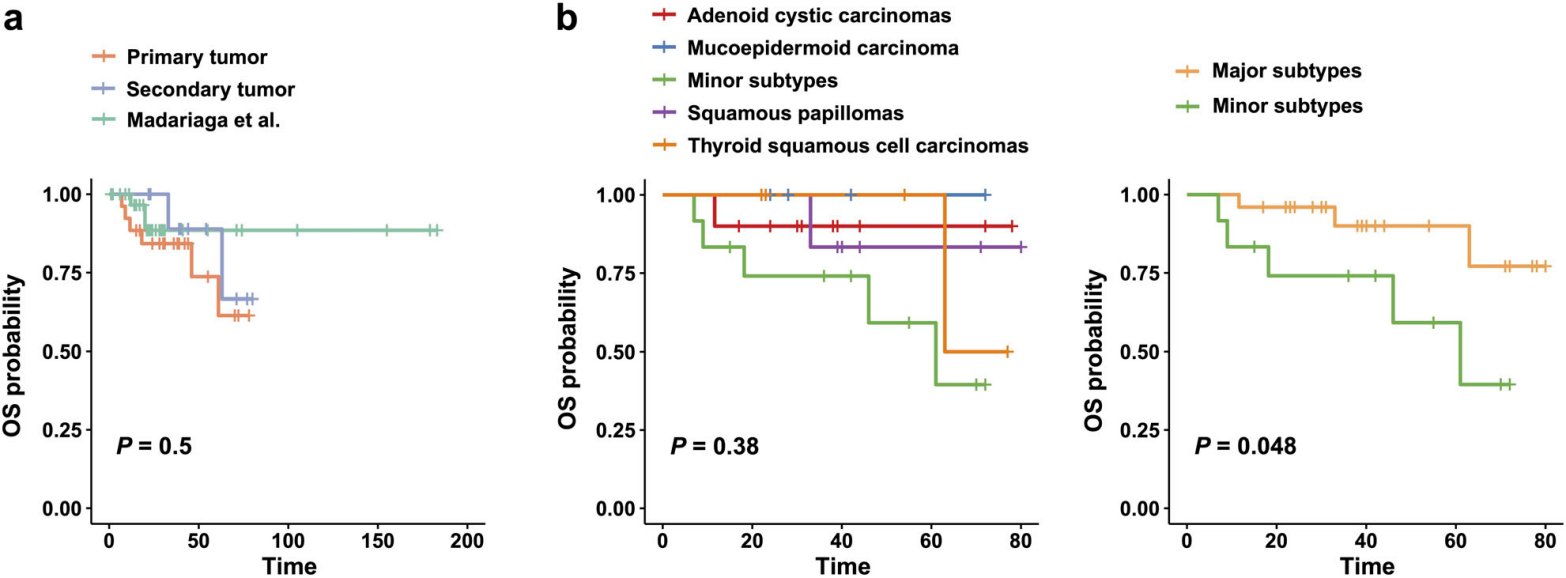
Long‐term prognosis of patients with tracheal tumor. (a) Comparison of long‐term prognosis between primary and secondary tracheal tumors. (b) Survival analysis on diverse histological subtypes (left panel), and differential survival results were observed between major and minor subtypes (right panel). OS, overall survival

A total of 37 patients with tracheal tumor were included in this study. Among these cases, 26 primary tracheal tumors were identified which were located in the extrathoracic trachea (9 of 26), middle trachea (9 of 26), and lower airway (8 of 26). Adenoid cystic carcinomas and mucoepidermoid carcinoma were major histological subtypes of primary tracheal tumor (Table 1), and minor subtypes included lung squamous cell carcinomas (*n* = 3), salivary glands (*n* = 3), neuroendocrine carcinoma (*n* = 2), acinic cell carcinoma (*n* = 1), carcinoid (*n* = 1), glomangioma (*n* = 1), and neurofibroma (*n* = 1). The remaining 11 advanced thyroid cancers were most often invading the upper trachea (7 of 11) and then the middle trachea (4 of 11), among which we found two histological types of squamous papilloma and thyroid squamous cell carcinoma (Table 1). No significant differences were found for the distribution of tumor locations between this study and a previous report (Figure S1a).^14^ To further estimate discrepancies between primary and secondary tracheal tumors, we analyzed the long‐term prognosis among two tumor origins and the data from a previous review.^2^ The results indicated a favorable prognosis for patients with tracheal tumors, and no significant differences were observed between primary and secondary tracheal tumors (Figure 1a). Subgroup analysis demonstrated that minor subtypes of primary tracheal tumor were associated with a poor prognosis (Figure 1b).

**TABLE 1:** Characteristics of cases included in the study

	*n*
Gender	
Male	21
Female	16
Age (years)	
≥60	17
<60	20
Tobacco	
Smoking	20
Non‐smoking	17
Tumor location	
Upper trachea	16
Middle trachea	13
Lower airway	8
Lumen obstruction	
≥50%	8
<50%	29
Tumor origin	
Primary (P)	26
Secondary (S)	11
Histology	
Adenoid cystic carcinomas (P)	10
Mucoepidermoid carcinoma (P)	4
Other subtypes (P)	12
Squamous papillomas (S)	6
Thyroid squamous cell carcinomas (S)	5
Complications	
Yes	6
No	31
ICU stay (days)	
1	25
>1	12
Minimal SpO2^a^	
≥95%	28
<95%	9
Ventilation and intubation	
ECMO	1
Tracheotomy	4
Cross‐field endotracheal intubation	24
Cross‐field endobronchial intubation	8

*Note*: Baseline characteristics of patients, features of tracheal tumors, postoperative complications, and anesthesia data.

Abbreviations: ECMO, extracorporeal membrane oxygenation; ICU, intensive care unit.

^a^ The minimal level of oxygen saturation during tracheal surgery.

### Airway selection for patients with tracheal tumor

**FIGURE 2:**
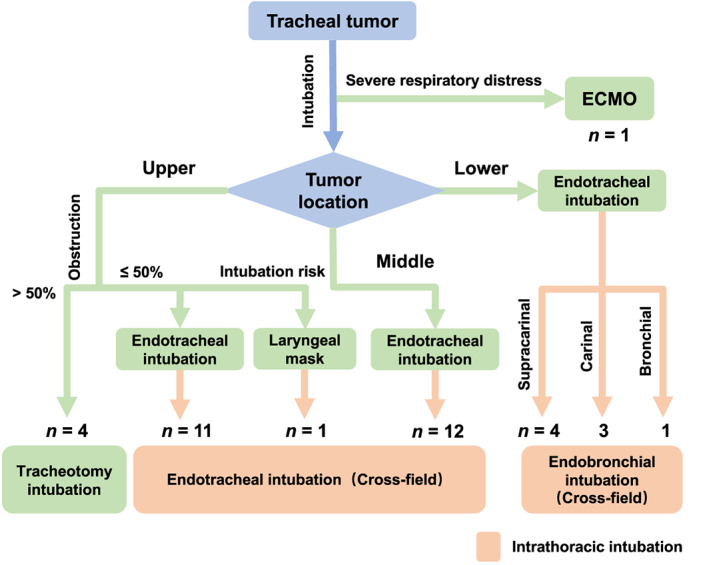
Diagram of airway management for tracheal surgery. Extracorporeal membrane oxygenation (ECMO) was recommended for the patient with severe respiratory distress; tracheotomy was performed when the tracheal tumor obstructed more than 50% of the upper lumen, and a laryngeal mask was used for the patient with intubation risk of tumor bleeding or shedding; cross‐field ventilation of intrathoracic intubation was conducted for the remaining cases

In this case series, we summarized the selection procedure of ventilation strategy for tracheal surgeries (Figure 2). For the case with severe respiratory distress, extracorporeal life support was used for intraoperative oxygenation. For patients with upper tracheal tumors, we chose preoperative tracheotomy intubation when more than 50% (*n* = 4) of the tumor was found to be obstructing the tracheal lumen. We first performed routine endotracheal intubation on other patients with tracheal tumors, except for the case in which there was an intubation risk of the tumor bleeding or shedding. We then applied cross‐field ventilation of intrathoracic intubation during the surgical process. Intrathoracically, endotracheal intubation was considered among cases with upper or middle tracheal tumors, and endobronchial intubation was conducted for the surgery of tumors in the lower airway. After circumferential dissection, one‐lung ventilation was processed for patients with lower tracheal tumors until the completion of end‐to‐end anastomosis. To further estimate the rationality of our selection procedure, we compared our results with a previous study,^11^ and similar airway selections were revealed between this study and the independent report (Figure S1b).

### Risk factors of intraoperative ventilation in tracheal tumor patients

Furthermore, we analyzed the association between ventilation strategies, patient characteristics and tumor features (Table 2). Our results demonstrated that airway selection correlated with tumor location, lumen obstruction, and tumor origin, which indicated the basis of selection strategies. Additionally, tobacco and tumor histology contributed towards a trend in this analysis, which suggested that the pathological properties of tumor could impact the final therapeutic decision. Notably, we found that different types of intraoperative ventilation correlated with the length of ICU stay, and known factors, including tracheotomy, use of ECMO, and carinal reconstruction, within two subgroups (tracheotomy/ECMO and cross‐field endobronchial intubation) contributed to ICU delay.

**TABLE 2:** Comparison of characteristics among subgroups of different ventilation strategies

	Tracheotomy/ECMO	Cross‐field endotracheal intubation	Cross‐field endobronchial intubation	*p‐*value
Gender				
Male	2	12	7	0.16
Female	3	12	1	
Age (years)				
≥60	3	11	3	0.79
<60	2	13	5	
Tobacco				
Smoking	3	10	7	0.07
Non‐smoking	2	14	1	
Tumor location				
Upper	4	12	0	<0.001
Middle	1	12	0	
Lower	0	0	8	
Lumen obstruction				
≥50%	5	1	2	<0.001
<50%	0	23	6	
Tumor origin				
Primary (P)	2	16	8	0.045
Secondary (S)	3	8	0	
Histology				
Adenoid cystic carcinomas (P)	2	6	2	0.09
Mucoepidermoid carcinoma (P)	0	2	2	
Other subtypes (P)	0	8	4	
Squamous papillomas (S)	3	3	0	
Thyroid squamous cell carcinomas (S)	0	5	0	
Complications				
Yes	2	3	1	0.33
No	3	21	7	
ICU days				
1	0	21	4	<0.001
>1	5	3	4	
Preoperative FEV1/FVC				
≥Median	1	14	2	0.16
<Median	4	10	6	
Intraoperative PaO2				
≥Median	3	13	2	0.4
<Median	2	11	6	
Minimal SpO2^a^				
≥95%	1	4	4	0.23
<95%	4	20	4	

Abbreviation: ICU, intensive care unit.

^a^ The minimal level of oxygen saturation during tracheal surgery.

Although the length of ICU stay resulted from ventilation selection, we attempted to discover other potential risk factors of ICU delay. We analyzed the number of events among subgroups using logistical regression, and the results indicated that a total of seven factors correlated with a higher risk of ICU delay (Table 3). Among these risk factors, a decline in oxygen saturation (SpO2 < 95%) during the surgical procedure was revealed to be an independent risk factor by the permutation test procedure (Methods).

**TABLE 3:** Risk factors of ICU delay based on a permutation test procedure for inference in logistic regression (methods)

	High/low risk of ICU delay	Univariate *p*‐value	Multivariate *p*‐value
Age (years)	≥60/<60	0.019	ns
Lumen obstruction	≥50%/<50%	0.009	ns
Histology	Nonsquamous papillomas/squamous papillomas	0.068	ns
FEV1/FVC	<Median/≥Median	0.021	ns
Intraoperative PaO2	<Median/≥Median	0.054	ns
Intubation	Nonendotracheal (CF)/endotracheal (CF)	0.001	ns
Minimal SpO2^a^	<95%/≥95%	0.001	0.021

Abbreviations: CF, cross‐field; ICU, intensive care unit; ns, nonsignificant.

^a^ The minimal level of oxygen saturation during tracheal surgery.

## DISCUSSION

Surgical interference is the preferred option for intratracheal mass, including primary and secondary tracheal tumors.^20^ Peri‐ and intraoperative airway management is critical to the success of complicated tracheal surgery, and our center is experienced at performing tracheal resection and reconstruction in patients with tracheal tumors. In this study, we reviewed and analyzed almost all cases who underwent surgical interference in our center. Most patients (32 of 37) underwent intraoperative airway transition with intrathoracic intubation, and selection strategies for intraoperative ventilation were demonstrated and corresponding indications in this case series.

For those patients with appropriate indications, VATS could be a therapeutic choice and the intercostal intubation is a relatively safe method.^21^ We applied VATS in one case with a middle tracheal tumor, for which we used the fifth intercostal space for the transition of intrathoracic intubation. For high‐risk patients with severe tracheal obstruction or poor pulmonary function, jet ventilation and ECMO are recommended measures for better oxygenation,^6^ which achieved a positive effect in one of our cases with severe respiratory distress. Notably, all upper tracheal tumors with extra‐luminal invasion were derived from advanced thyroid carcinoma, and a laryngeal mask is recommended to avoid the potential risk of the tumor bleeding or shedding. In addition to the surgical process and intubation strategy, more attention should be given to the ventilation efficacy when performing intrathoracic endotracheal or endobronchial intubations, and appropriate ventilation should also be considered.

Primary tracheal tumors are rare, and only few studies have previously been reported. Almost all surgical techniques for tracheal tumors have been summarized and discussed in previous studies,^8^, ^22^, ^23^ but related airway management during the surgical procedure was seldom estimated in these reports. The description of intraoperative airway management was first introduced in a previous case report,^24^ which details the ventilation strategy for a patient with an upper tracheal tumor. Additionally, Schneider et al. reported their surgical experiences among 14 cases with tracheal tumors, and first recommended radical tracheal resection and reconstruction as the preferred choice for primary malignant tracheal tumors.^1^ A favorable long‐term prognosis was indeed observed among patients with primary tracheal tumor in this case series. For cases with advanced thyroid cancer invading the upper airway, Nakao et al. reported a favorable local control rate for patients who underwent radical surgery,^25^ and our study also found that these patients benefited from surgical treatment and achieved long‐term survival. In summary, complicated tracheal surgery plays a decisive role in tracheal tumor therapy, which further emphasizes the importance of team collaboration between surgeons and anesthesiologists.

Overall, close cooperation between the surgeon and anesthesiologist plays an important role in the successful application of tracheal resection and reconstruction, and surgical treatment combined with appropriate airway management is a safe procedure at an experienced center. The selection of an appropriate airway during the surgical process is a matter of focus because too few cases have been reported in randomized studies. It is hoped that this case series will provide further evidence for this complicated therapeutic procedure.

## CONFLICT OF INTEREST

No potential conflicts of interest are disclosed.

## Supporting information


**Figure S1**. Comparison of characteristics and ventilation strategies between this study and other independent reports. (A) The distribution of airway locations of tracheal tumors between this study and Kutzner et al.'s report. (B) Proportions of different ventilation strategies between this study and the study by Schieren et al.
