# Supplementary figures and images for: Intraoperative airway management for patients with tracheal tumors: A case series of 37 patients
Source: Thorac Cancer. 2021 Oct 9;12(22):3046–52. doi: 10.1111/1759-7714.14181 (PMC8590894; doi:10.1111/1759-7714.14181)

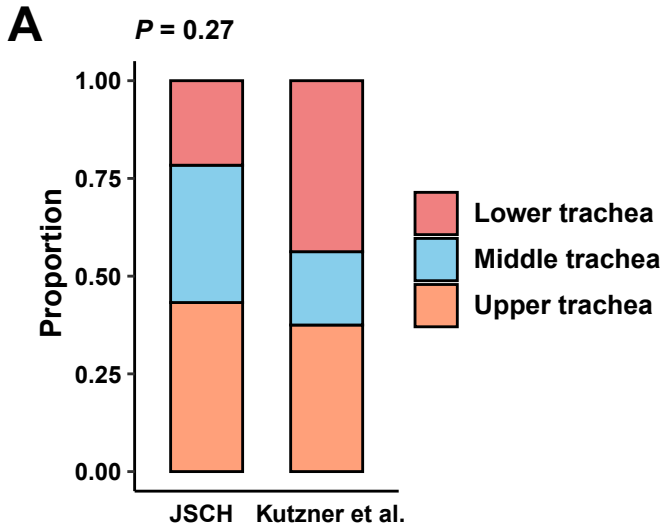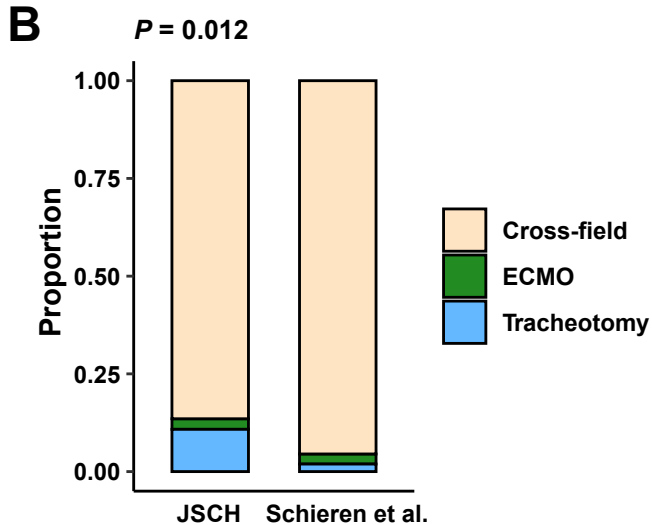

Supplement: Supplementary file 1 — Figure S1. Comparison of characteristics and ventilation strategies between this study and other independent reports. (A) The distribution of airway locations of tracheal tumors between this study and Kutzner et al.'s report. (B) Proportions of different ventilation strategies between this study and the study by Schieren et al. [file TCA-12-3046-s001.pdf]
